# Supplementary material for: Gut microbiota profiles of treatment-naïve adult acute myeloid leukemia patients with neutropenic fever during intensive chemotherapy
Source: PLoS One. 2020 Oct 28;15(10):e0236460. doi: 10.1371/journal.pone.0236460 (PMC7592783; doi:10.1371/journal.pone.0236460)
Supplement: S1 Table — Statistical corrections for multiple comparisons were performed using the original false discovery rate (FDR) method of Benjamini-Hochberg with desired false discovery rate (Q) of 0.05. (DOCX) [file pone.0236460.s001.docx]

**S1 Table. Relative abundance at genus level of phyla with a relative abundance over 10%.** Statistical corrections for multiple comparisons were performed using the original false discovery rate (FDR) method of Benjamini-Hochberg with desired false discovery rate (Q) of 0.05.

| Phylum | Genus | Mean difference  (q value, individual *P* value, discovery) | | |
| --- | --- | --- | --- | --- |
|  |  | Pretreatment  vs.  Febrile neutropenia | Pretreatment  vs.  BM recovery | Febrile neutropenia  vs.  BM recovery |
| Firmicutes | *Enterococcus* | 20.2  (0.0002, <0.0001, yes) | 15.4  (0.0138, 0.0092, yes) | -4.7  (0.4212, 0.4212, no) |
|  | *Streptococcus* | 0.7  (0.8824, 0.8824, no) | 2.6  (0.8824, 0.6479, no) | 1.9  (0.8824, 0.7438, no) |
|  | *Blautia* | -1.8  (0.9200, 0.6997, no) | -2.4  (0.9200, 0.6703, no) | 0.6  (0.9200, 0.9200, no) |
|  | *Veillonella* | -0.8  (0.8572, 0.8572, no) | 2.1  (0.8572, 0.7223, no) | 2.9  (0.8572, 0.6191, no) |
| Bacteroidetes | *Bacteroides* | -9.4  (0.4684, 0.1561, no) | -5.1  (0.5895, 0.5144, no) | 4.3  (0.5895, 0.5895, no) |
|  | *Parabacteroides* | -1.4  (0.8268, 0.8268, no) | 4.3  (0.8268, 0.5784, no) | 5.8  (0.8268, 0.4687, no) |
| Proteobacteria | *Sutterella* | -2.4  (0.9167, 0.5646, no) | -2.4  (0.9167, 0.6112, no) | -0.1  (0.9982, 0.9982, no) |
|  | *Escherichia* | -11.2  (0.0191, 0.0064, yes) | -11.8  (0.0237, 0.0158, yes) | -0.5  (0.9073, 0.9073, no) |
|  | *Klebsiella* | 1.7  (0.6757, 0.6757, no) | 4.8  (0.6757, 0.3139, no) | 3.2  (0.6757, 0.5185, no) |
