# Supplementary material for: Gut microbiota profiles of treatment-naïve adult acute myeloid leukemia patients with neutropenic fever during intensive chemotherapy
Source: PLoS One. 2020 Oct 28;15(10):e0236460. doi: 10.1371/journal.pone.0236460 (PMC7592783; doi:10.1371/journal.pone.0236460)
Supplement: S2 Table — (DOCX) [file pone.0236460.s002.docx]

**S2 Table. The numbers of OTUs per sample.**

| Patient code | Pretreatment | Febrile neutropenia | Bone marrow recovery |
| --- | --- | --- | --- |
| P1 | 182 | 189 | NA |
| P2 | 223 | 176 | NA |
| P3 | 193 | NA | 35 |
| P4 | 145 | 72 | 91 |
| P5 | 143 | 105 | NA |
| P6 | 251 | 170 | 122 |
| P7 | 213 | 108 | NA |
| P8 | 129 | NA | 123 |
| P9 | 265 | 102 | 279 |
| P10 | 107 | NA | NA |

NA: Not available
