# Supplementary material for: Gut microbiota profiles of treatment-naïve adult acute myeloid leukemia patients with neutropenic fever during intensive chemotherapy
Source: PLoS One. 2020 Oct 28;15(10):e0236460. doi: 10.1371/journal.pone.0236460 (PMC7592783; doi:10.1371/journal.pone.0236460)
Supplement: Attachment — Submitted filename: Response to reviewers.docx [file pone.0236460.s003.docx]

**Response to reviewers**

**Reviewer #1:** The authors present a report in which they follow the microbiological flora ny NGS sequencing of bacterial DNA/rRNA in the guts of patients with AML . They compare finds pretreatment , the time of neutropenic fever (F/N) as well as upon recovery. They make some observations concerning the changes noted; in particular, they seem to show that bacterial diversity declines between pre-treatment and F/N

Comments

1. The abstract and the entire paper suffer from severe problems with grammar and syntax; these need to be addressed, most likely by a native English speaker.

**Response: The manuscript has already been revised by English language team.**

2. Abstract. This needs to be sufficiently re-worked to give a better idea to the reader of the quantitative findings. Further no mention is made of antibiotic usage, particularly prophylaxis, which could obviously affect the results.

**Response: Addition of quantitative findings and usage of antibiotics have been addressed.**

3. The Introduction should state what advantage the sequencing technology has over culturing methods to determine gut bacterial diversity.

**Response: The advantage of sequencing technology over traditional culturing methods has been addressed.**

4. Is the fact that all patients had invasive pulmonary aspergillosis unusual ( or so that is stated in the text, but not related in the table)?

**Response: Only 3 patients had invasive pulmonary aspergillosis. I have rewritten this statement into " Three patients (33.3%) were defined as microbiologically significant with invasive pulmonary aspergillosis confirmed by typical radiologic finding and serum galactomannan (indicated as patient's code: P4, P6 and P10)".**

5. What does 3 patients … microbiologically defined mean?

**Response: I have rewritten this statement into "Three patients (33.3%) were defined as microbiologically significant with invasive pulmonary aspergillosis confirmed by typical radiologic finding and serum galactomannan (indicated as patient's code: P4, P6 and P10)".**

6. The pretreatment neutrophil count seemed remarkably homogeneous for a group of patients with AML. The AML characteristic should be provided.

**Response: I have provided the AML patients' characteristic in Table 1. The median of pretreatment absolute neutrophil count was 2.85 x 10^9^ /L (1.42-7.67) with the range of 1.42 x 10^9^ /L (minimum) to 7.67 x 10^9^ /L (maximum).**

7. Table 2 what do the numbers in the leftmost column represent?

**Response: The leftmost column in table 2 represent the name of each bacterial phyla and next column is the percentage of microbial relative abundance among five most abundant stool bacterial phyla.**

8. In the discussion section the authors really don't give a good explanation of why the flora of the gut changed despite the lack of use of antibacterial prophylaxis (and before any specific therapy for fever neutropenia was given)

**Response: I have explained more detailed on the changes of gut microbiota in the discussion section. (We hypothesized that even the first cycle of intensive chemotherapy, which would contribute to the NF, and without antibiotic prophylaxis might reveal evidence of changes in intestinal bacteria flora as all of febrile neutropenia patients require empirical treatment with broad spectrum antibiotics. Gut barrier is a vulnerable site of injury after receiving highly intensive chemotherapy and resulting microbiota pattern disruption. The antibiotic therapy may eradicate particular taxa which affect host immunity and the chemotherapy can extensively damage the gut barrier, the neutropenia itself also potentially affecting the intestinal bacteria of the patients.)**

**Reviewer #2:** The authors present a study examining the bacterial composition of stool samples collected from patients undergoing initial induction chemotherapy treatment for AML. Three time points were analyzed from 10 patients, with 100% and 90% of samples successfully analyzed at baseline and at onset of febrile neutropenia, and 50% of samples analyzed at bone marrow recovery. The methodologies reported are appropriate and the manuscript is overall well-written. I have the following comments:

- The study overall is fairly limited. The sample size is only 10 patients, with significant drop-off of samples collected at the 3rd time point. Such a small sample size doesn't allow for exploration of microbiome associations with different clinical outcomes. Larger retrospective cohorts have already been published, and it is a but unclear what this current study adds to the field.

**Response: This study conducted solely treatment-naïve AML patients who homogenously experienced the first episode of neutropenic fever and currently not received antimicrobial prophylaxis. Unfortunately, the limitation of clinical correlation cannot be eliminated but to point out the microbial composition changes even in the first dose of AML treatment which microbiota diversity remained constant despite recovery of bone marrow. Moreover, the study also expand the dataset of gut microbiota profiles in adult Asian ethnicity with AML.**

- Comparisons of microbiome parameters (abundances of taxa or diversity) should be adjusted for multiple comparisons, using for example the Benjamini-Hochberg method. They currently don't appear to be adjusted.

**Response: In terms of correction of multiple comparisons, Benjamini-Hochberg method has been introduced to the manuscript.**

- Conveying bacterial abundances using tables is probably not as effective as graphically. Consider preparing stacked bar graphs for individual samples.

**Response: I have converted table 3 to the stacked bar graph for individual sample and this table has been moved to supplementary data (Table S1).**

- Data underlying the findings described are not fully available in the manuscript, supplementary materials, or in an online repository.

**Response: All of the sequencing results had been provided as an online accession of Sequence Read Archrive (SRA), as BioProject number PRJNA661595 (**[**https://www.ncbi.nlm.nih.gov/Traces/study/?acc=PRJNA661595**](https://www.ncbi.nlm.nih.gov/Traces/study/?acc=PRJNA661595)**),**

**and supplementary material has been added.**

- Consider asking a native English speaker to edit the manuscript.

**Response: The manuscript has already been revised by English language team.**
